# Multi‐tissue multi‐omic trans QTL mapping identified novel causal and druggable targets and gene‐gene interactions

**DOI:** 10.1002/alz.092051

**Published:** 2025-01-03

**Authors:** Carlos Cruchaga

**Affiliations:** ^1^ Washington University in St. Louis, School of Medicine, St. Louis, MO USA

## Abstract

**Background:**

The integration of quantitative trait loci (QTLs) with disease genome‐wide association studies (GWAS) has proven successful at prioritizing candidate genes at disease‐associated loci. Most of QTL studies are focusing on expression QTLs in plasma and brain and cis‐signals.

**Method:**

Here we analyzed a large proteomic (Somalogic 7K) and metabolomic (Metabolon HD4) CSF (n = 3, 000) and plasma (African (AFR, N = 400) and European (EUR, N = 2,300) ancestry, respectively) to identify novel QTLs. We integrated with FUSION, colocalization and Mendelian Randomization approaches to identify novel causal and druggable targets.

**Result:**

In CSF we identified 2,316 significant pQTLs (1,247 in *cis* and 1,069 in *trans*) for 1,960 proteins, of which 1,228 were not observed in plasma. For metabolomics we identified 219 independent associations (59.8% novel) for 144 CSF metabolites. In plasma, we identified 2,400 pQTL in EUR and 881 pQTL in AFR. In EUR and AFR metabolomics, we reported 403 and 60 metabolites with at least one significant QTL, respectively. Approximately 40% of these findings were novel associations compared to previous studies Through PWAS, we identified 473 CSF proteins associated with AD risk. MR prioritized 40 proteins as causal and colocalization identified 158 proteins that share genetic etiology. 42 of these overlap between at least two of these methods and are enriched in immune and lysosomal pathways. Multiple (including PILRA, PRSS8, and SIRPA) represent novel candidate proteins. We also identified novel regulators for TREM2 including TGBR2 and NECTIN2, which represent novel therapeutic targets for AD.

**Conclusion:**

We have developed the largest CSF proteomics and metabolomics data. Through a rigorous approach combining three methods, we have identified high‐confidence proteins involved in AD that confirm previously reported candidate genes and prioritize new ones at GWAS loci. Our findings offer insights into Alzheimer’s disease biology that were missing when using plasma analyses, supporting the development of tissue‐specific proteomics databases in neurologically‐relevant tissues.

